# Avulsion Fracture of the Lesser Trochanter in a Young Adolescent

**DOI:** 10.7759/cureus.116228

**Published:** 2026-09-14

**Authors:** Hafid Talha, Abdelkhalek Hamoutahra

**Affiliations:** 1 Pediatric Surgery Department, Moulay Ali Cherif Regional Hospital Center, Errachidia, MAR; 2 Laboratory of Health Sciences, Faculty of Medicine and Pharmacy, Moulay Ismail University of Meknes, Errachidia, MAR; 3 Pediatric Surgery Department, Faculty of Medicine and Pharmacy of Laayoune, Moulay Hassan Ben Mahdi Regional Hospital, Laayoune, MAR

**Keywords:** adolescent, athletic injuries, avulsion fracture, conservative treatment, lesser trochanter

## Abstract

Avulsion fracture of the lesser trochanter occurs when the iliopsoas tendon detaches from its bony insertion and is more commonly associated with sports-related injuries. We report the case of a 14-year-old male who developed acute right groin pain and functional impairment after a triple jump. Examination revealed pain during right hip extension. Anteroposterior pelvic radiography demonstrated an avulsion fracture of the right lesser trochanter, which was confirmed by CT. The patient was treated conservatively with rest and reduced weight-bearing on the affected limb. His pain resolved within a few weeks, and he resumed sporting activity six months after the injury. At the two-year follow-up, radiography showed complete union of the lesser trochanteric fracture. Although rare, an avulsion fracture of the lesser trochanter should be considered in adolescents presenting with acute groin pain after sports activity. Plain pelvic radiography is usually sufficient to establish the diagnosis, while conservative treatment generally results in satisfactory healing in most uncomplicated cases.

## Introduction

The iliopsoas muscle inserts into the lesser trochanter, an apophysis that ossifies late [1]; the tendon of the iliopsoas muscle itself is more resistant than the lesser trochanter [2]. Groin injuries represent up to 5% of all adolescent sports injuries [3], and lesions of the lesser trochanter remain uncommon, with isolated fractures accounting for less than 1% of injuries encountered in sports medicine practice [4]. Given the rarity of such traumatic lesions, they pose considerable diagnostic challenges, as most of the time, the child is sent home without a radiograph, and the fracture is recognized only later when no improvement is noted [5]. Non-operative care is recommended, except for symptomatic nonunion or painful exostosis [5]; arthroscopically assisted screw fixation has been proposed for fragments displaced by more than 2 cm, associated with hip flexor weakness and ischio-femoral impingement [4]. A pooled analysis of pelvic avulsions at all sites has reported a higher rate of return to the pre-injury level after surgery than after conservative treatment [1]. We report here the case of a 14-year-old boy who sustained an isolated avulsion fracture of the lesser trochanter during a triple jump, which was managed conservatively and showed a good recovery in mobility and function.

## Case presentation

A 14-year-old boy was evaluated after injuring his pelvis sustained during the triple jump. He reported right groin pain and weakness of the right lower limb afterward. The injury occurred during the take-off phase of the triple jump, as he pushed off forcefully with his right leg. On physical examination, movement of the right hip into extension reproduced the pain; he had no skin lesions or deformities of the groin or right limb. An anteroposterior pelvic radiograph showed an avulsion fracture of the right lesser trochanter (Figure 1), and CT confirmed the avulsed fragment (Figure 2).

**Figure 1:**
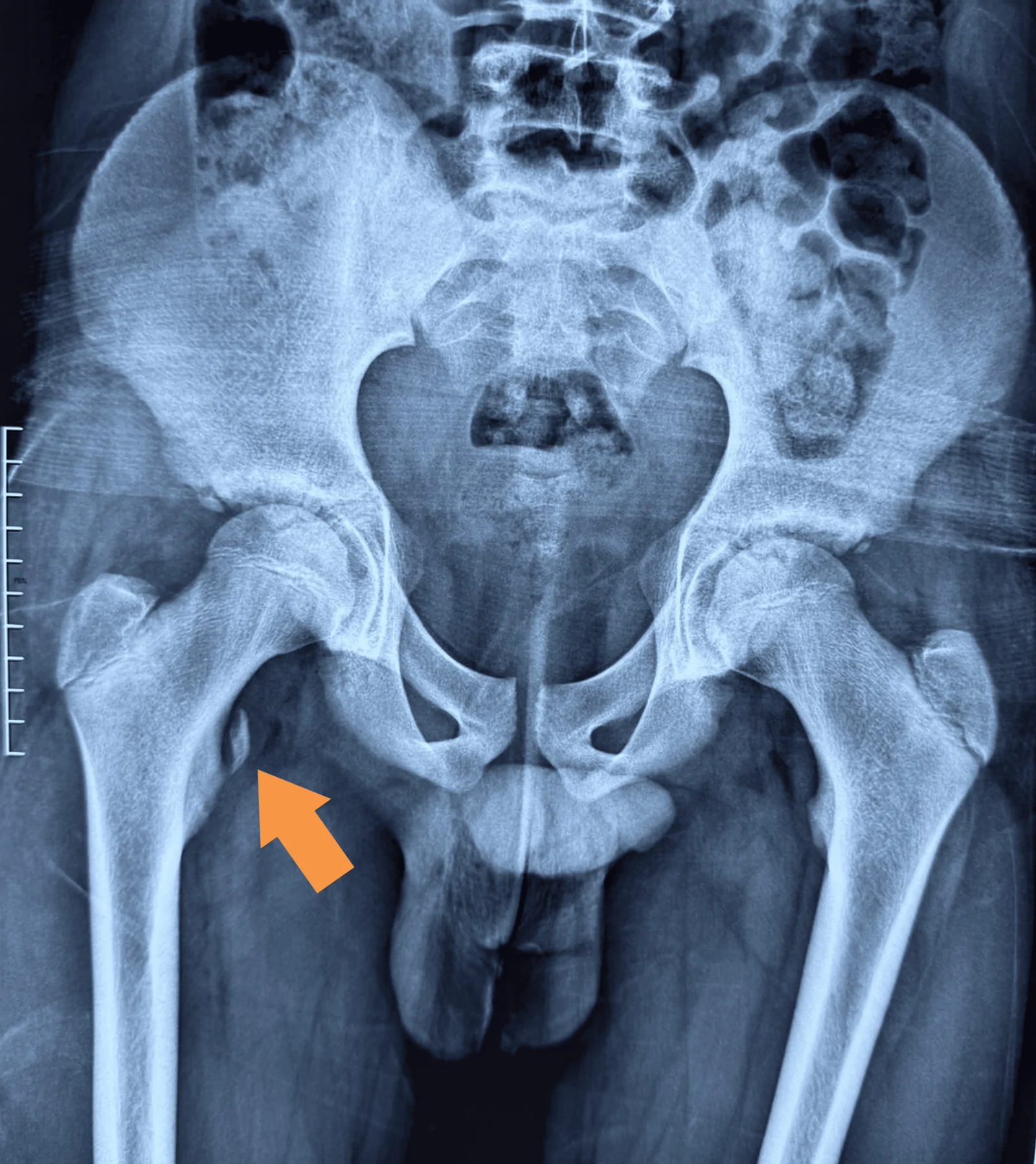
Anteroposterior pelvic radiograph at initial presentation The image shows an avulsion fracture of the right lesser trochanter (arrow)

**Figure 2:**
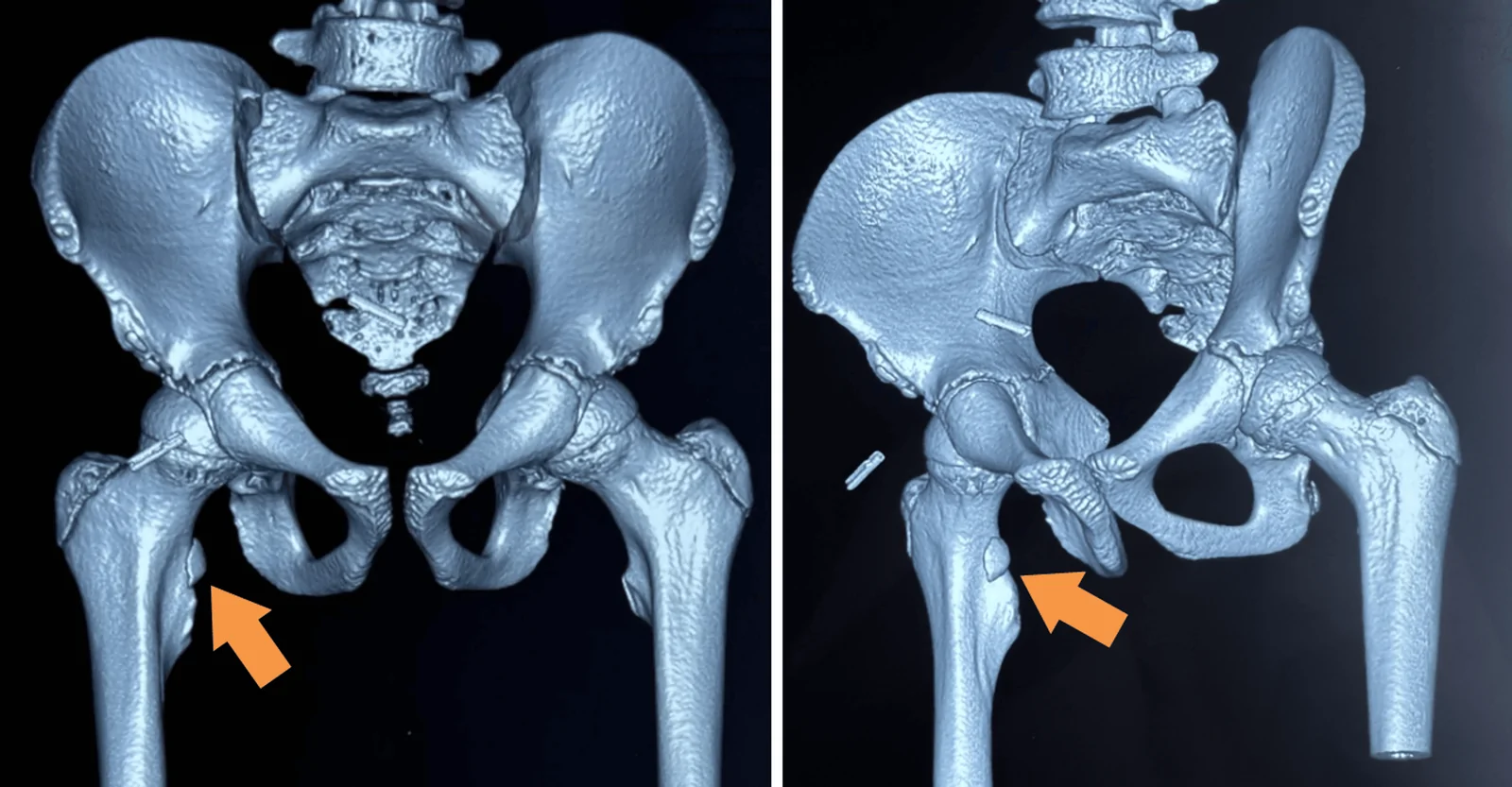
Three-dimensional CT reconstruction of the pelvis Anterior (A) and lateral (B) views showing the avulsed right lesser trochanteric fragment (arrows) CT: computed tomography

The patient was hospitalized for one week (D0-D7) and managed conservatively with rest, nonsteroidal anti-inflammatory drugs, analgesics, and partial weight-bearing on the right lower limb using crutches for five weeks (D0-D35). He showed a favorable clinical course, with complete resolution of pain within a few weeks (D21) and a return to sports six months after the injury (6M). A radiograph obtained at the two-year follow-up (2Y) demonstrated complete union of the right lesser trochanter (Figure 3).

**Figure 3:**
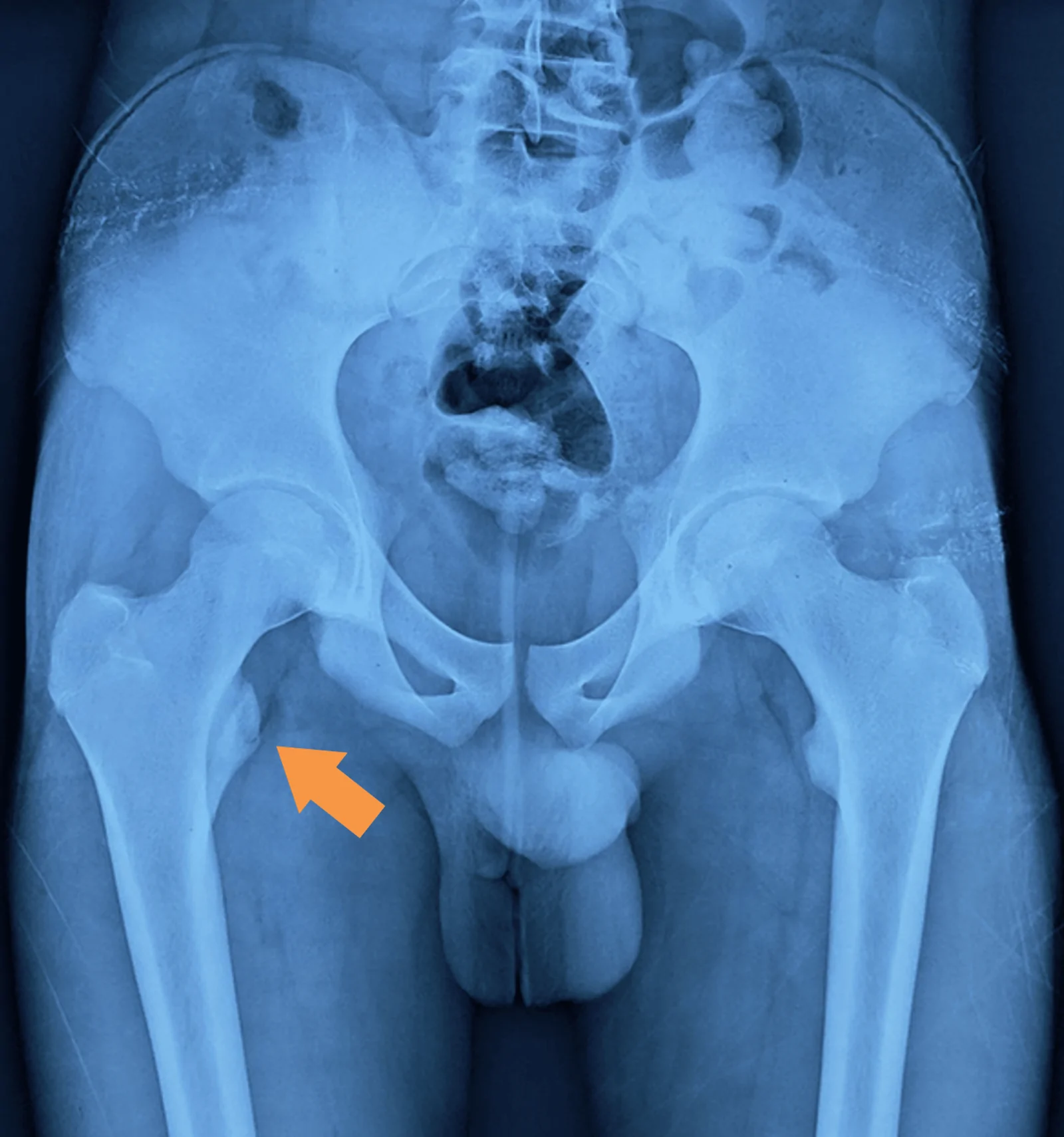
Anteroposterior pelvic radiograph at the two-year follow-up The image shows the complete union of the right lesser trochanter (arrow)

## Discussion

Isolated avulsion of the lesser trochanter is a rare occurrence; it accounts for less than 3% of all pelvic avulsion fractures and less than 1% of hip injuries in adolescents [6]. Jonasch et al. identified five trochanteric avulsions, including both greater and lesser trochanteric avulsions, among 263,166 injured children aged 14 years and younger over 10 years [5]. Hösli et al. reported three cases in a study conducted over two decades [7]. In a study conducted by Di Maria et al., a lesser trochanter avulsion was identified in 9% of 453 cases of pelvic avulsions [1]. This lesion was mostly described in patients aged between eight and 16 years, with a peak at 14 years [8]. The main mechanism of such lesions most often involves a sudden eccentric contraction of the iliopsoas muscle during acceleration or deceleration [4], and less frequently, a passive stretch of the iliopsoas muscle [4]. Most cases occur during sprinting or kicking motions. Two of the three boys in Tahir et al. heard a pop and could not finish a sprint at school; the third sustained the injury after a fall from a horse [2], while Pallis et al. reported a 14-year-old who was kicking a ball [6]. Outside sporting circumstances, a tonic-clonic seizure may cause the same fracture [9].

The clinical presentation can be variable, including antalgic gait, groin tenderness, an inability to perform active straight-leg raising, and the limb lying in slight flexion with external rotation or in flexion, adduction, and internal rotation [8]. The inability to lift the thigh in the sitting position, known as the Ludloff sign, is typical. The pitfall in the emergency room is to make a diagnosis of groin strain without obtaining a radiograph [5]. Anteroposterior pelvic radiograph confirms the diagnosis and assesses the amount of displacement [5,6]. CT and MRI do not seem to have any influence on treatment [5]. MRI can be helpful when the avulsed bone is not yet ossified [5]. In an adult, the same fracture should raise suspicion for a metastasis [2]. McKinney et al. classified these avulsions according to displacement: type 1, non-displaced; type 2, displaced by 2 cm or less; type 3, displaced by more than 2 cm; and type 4, symptomatic nonunion or painful exostosis. Nonoperative treatment is recommended for types 1 to 3, and surgery is discussed only for type 4 [10]. Migration remains limited because of the persistent attachment of the periosteum to the avulsed fragment [2].

The common management protocols include withdrawal from sport, protected weight bearing, and analgesia. Tahir et al. used non-weight-bearing on crutches for two weeks, repeated the radiograph, then began mobilization and strengthening once pain had settled at three weeks, allowed full weight-bearing at six weeks, and found hip flexion strength equal to the opposite side at 12 weeks [2]. Pallis et al. delayed partial weight-bearing until week four [6]. Ruffing et al. permitted partial weight-bearing depending on tolerance from the start [5]. In Volpi et al.'s study, 17 of 30 patients were weight-bearing as tolerated from the start, and physiotherapy was prescribed in 18 patients, with a mean treatment duration of 30.7 days and a mean return to sport of 78.3 days [4]. Sport is avoided until healing, which may take up to 12 weeks [2]. Ruffing et al. maintained that these fractures should be treated nonoperatively [5].

Khemka et al. used fixation in three adolescents with displacement exceeding 2 cm and either nonunion, loss of flexion strength, or ischio-femoral impingement [11]. Otto et al. obtained an excellent result in two adolescents with retrograde fixation, with return to sport at 10 to 12 weeks (Table 1) [12]. Fixation carries some risks, such as narrowing of the ischio-femoral space caused by the screw used [4]. Both open and arthroscopic procedures expose the patient to heterotopic ossification [13,14], and a medial approach may cause injury to the femoral vessels or the anterior branch of the obturator nerve [2]. The follow-up period is variable according to different studies, with the longest being a 4.9-year mean in Ruffing et al. [5]. Di Maria et al. reported a 95% return to the pre-injury level after surgery, compared with 79% without surgery, across all pelvic sites [1]. Only 40 of their 453 patients had a lesser trochanter lesion, of whom 35 were treated conservatively [1].

**Table 1:** Published reports about the management of avulsion fracture of the lesser trochanter in adolescents (compared with our case) HHS: Harris Hip Score

Study	Number of patients	Age (years)	Displacement	Management	Return to sport	Outcome
Khemka et al. (2014) [11]	3	15-16	All greater than 2 cm	Arthroscopically assisted fixation using K-wire, cancellous screw, suture anchor; weight-bearing as tolerated for 4 weeks, then physical therapy	10-12 weeks	Full recovery
Otto et al. (2019) [12]	2	12 and 16	-	Retrograde open reduction and fixation through an anterior approach	-	Excellent outcome
Volpi et al. (2021) [4]	30	14.2 (7.7-17.5)	Mean 5.1 mm (0-17)	Conservative; sport stopped in all; weight-bearing as tolerated in 17 (57%); physical therapy in 18 (60%). Mean treatment duration: 30.7 days	Mean: 78.3 days	No complications
Ruffing et al. (2018) [5]	5	13.8 (13-15)	2 type II, 3 type III	Conservative; analgesia and partial weight-bearing with crutches. Full weight-bearing at 6 weeks	At competitive level	HHS 100/100 at mean 4.9 years, strength grade: 5
Present case	1	14	1 cm	Conservative. Rest and offloading of the injured limb	At 6 months	Pain resolved within a few weeks

## Conclusions

Isolated avulsion fracture of the lesser trochanter is rare but should be considered when an adolescent develops acute groin pain after a sudden sporting movement. In this case, radiography followed by CT established the diagnosis, and conservative management resulted in resolution of pain, return to sport, and complete radiographic union over two years. Early recognition is important to avoid misdiagnosis as a simple groin strain and to ensure appropriate activity restriction and clinical and radiographic follow-up.
